# Cephalic Vein Puncture in CIED Implantation: The Emerging Standard and Its Clinical Implications

**DOI:** 10.1002/clc.70005

**Published:** 2024-08-22

**Authors:** Mustafa Mansoor, Ibrahim Manzoor, Muhammad Ahmed

**Affiliations:** ^1^ Islamic International Medical College Islamabad Pakistan

Dear Editor,

The use of cardiac implantable electronic devices (CIRDs) has seen a significant rise in recent years. The European Heart Rhythm Association (EHRA) reported a 20% increase in pacemaker (PM) implantations and a 44% increase in implantable cardioverter‐defibrillator (ICD) over a 10‐year period in its member countries, prompting the need for safe, efficient, and simple‐to‐master techniques for establishing venous access [1]. The latest guidelines from the EHRA recommend cephalic vein access, commonly done via cephalic vein cut‐down (CVC), for CIRD implantation. However, the greater skill and training required for CVC, coupled with anatomical challenges, often lead to the usage of alternative subclavian venous access (SVC) in patients initially approached via CVC, increasing adverse events [2]. To address this, a modified Seldinger technique has been described recently, offering the potential for an easier‐to‐learn method with decreased complexity, promising higher success rates and fewer adverse events.

To assess the efficacy and safety of cephalic venous puncture (CVP) compared to SVP for CIED implantation, Weidauer et al. conducted a study [3]. In a setting where most surgeons lacked prior training in cephalic vein access, CVP was mandated for all procedures. The researchers employed the modified Seldinger technique for CVP, involving initial cephalic vein puncture followed by guidewire‐facilitated catheter or sheath insertion. This less invasive approach avoided the need for direct subclavian vein puncture using a large‐bore needle. The study involved 229 consecutive patients receiving a CIED. Among these patients, 61 were implanted using primary or bail‐out SVP, while 168 patients underwent primary cephalic vein preparation with CVP when feasible. Results showed successful implantation of at least one lead in 90% of CVP patients, with complete lead implantation in 72.6%. There were no significant differences in procedure time, fluoroscopy use, or radiation dose between the two groups. Importantly, none of the 122 patients with solely CVP lead implantation developed pneumothorax, compared to 7.5% in the SVP group with at least one lead through SVP. Hence, showing that changing the mandatory primary venous access for CIED from a subclavian puncture to the cephalic vein can be achieved without compromising procedure times or success rates.

The study's robust design and consistent findings significantly contribute to establishing CVP as a potential standard procedure for CIED implantation. In this context, the axillary vein puncture (AVP) approach has emerged as a viable alternative, demonstrating high success rates, low complication rates, reduced procedural times, and lower radiation exposure [4]. Direct visualization of the vessel during puncture is facilitated by the axillary approach. Furthermore, ultrasound‐guided axillary access (USAA) proves advantageous for patients with challenging thoracic anatomy, such as obesity, extreme thinness, or anticoagulant therapy, as it enables faster and safer cannulation. The axillary approach also mitigates the risk of mechanical stress on implanted leads and pneumothorax [5]. Therefore, a comprehensive comparison between traditional CVP and AVP would offer valuable insights into their respective strengths and weaknesses and should be explored in further studies. Considering AVP's current standing as the more effective method, a direct comparative analysis could uncover critical differences to inform optimal clinical practice. Furthermore, the study's design limitations impede a direct comparison of CVP and SVP success rates. Since SVP is predominantly employed as a backup option, a robust comparison between the two methods is challenging. Moreover, the non‐randomized design, with patient allocation based on vein anatomy or failed CVP, introduces potential biases. To rectify these shortcomings, future research should prioritize randomized controlled trials or employ propensity score matching to ensure comparable patient groups. Incorporating detailed patient characteristics, including vein anatomy and other relevant factors, will enhance our comprehension of patient selection criteria for CVP and SVP. By addressing these limitations, future studies can deliver more comprehensive insights into the relative efficacy of CVP, SVP, and AVP.

## Author Contributions

Mustafa Mansoor came up with the concept, design, data acquisition, analysis, and interpretation of the study. Ibrahim Manzoor helped in writing and provided supportive ideas. Muhammad Ahmed helped in editing and reviewing.

## Conflicts of Interest

The authors declare no conflicts of interest.

## Data Availability

Data sharing is not applicable to this article as no data sets were generated or analyzed during the current study.
